# Encapsulation of β-Carotene by Emulsion Electrospraying Using Deep Eutectic Solvents

**DOI:** 10.3390/molecules25040981

**Published:** 2020-02-22

**Authors:** Ahmet Ozan Basar, Cristina Prieto, Erwann Durand, Pierre Villeneuve, Hilal Turkoglu Sasmazel, Jose Lagaron

**Affiliations:** 1 Novel Materials and Nanotechnology Group, IATA-CSIC, 46980 Valencia, Spain; ozanahmetbasar@gmail.com; 2 R&D Department, Bioinicia S.L., 46980 Valencia, Spain; 3 CIRAD, UMR IATE, F-34398 Montpellier, France; erwann.durand@cirad.fr (E.D.); pierre.villeneuve@cirad.fr (P.V.); 4 IATE, Univ Montpellier, CIRAD, INRA, Montpellier SupAgro, F-34398 Montpellier, France; 5 Department of Metallurgical and Materials Engineering, Atilim University, 06830 Ankara, Turkey; hilal.sasmazel@atilim.edu.tr

**Keywords:** deep eutectic solvents, emulsion electrospraying, encapsulation

## Abstract

The encapsulation β-carotene in whey protein concentrate (WPC) capsules through the emulsion electrospraying technique was studied, using deep eutectic solvents (DES) as solvents. These novel solvents are characterized by negligible volatility, a liquid state far below 0 °C, a broad range of polarity, high solubilization power strength for a wide range of compounds, especially poorly water-soluble compounds, high extraction ability, and high stabilization ability for some natural products. Four DES formulations were used, based on mixtures of choline chloride with water, propanediol, glucose, glycerol, or butanediol. β-Carotene was successfully encapsulated in a solubilized form within WPC capsules; as a DES formulation with choline chloride and butanediol, the formulation produced capsules with the highest carotenoid loading capacity. SEM micrographs demonstrated that round and smooth capsules with sizes around 2 µm were obtained. ATR-FTIR results showed the presence of DES in the WPC capsules, which indirectly anticipated the presence of β-carotene in the WPC capsules. Stability against photo-oxidation studies confirmed the expected presence of the bioactive and revealed that solubilized β-carotene loaded WPC capsules presented excellent photo-oxidation stability compared with free β-carotene. The capsules developed here clearly show the significant potential of the combination of DES and electrospraying for the encapsulation and stabilization of highly insoluble bioactive compounds.

## 1. Introduction

The encapsulation of bioactive compounds has emerged as an essential technology in the design of functional foods or active packaging applications [1,2,3], due to the high sensitivity of these compounds to different physico-chemical factors (temperature, oxygen, humidity, pH, among others), which produce their degradation, loss of nutritional value, and consequently loss of bioavailability [4]. Carotenoids, and concretely β-carotene, are a good example of this kind of challenging bioactive compounds. In addition to the previous challenge, most of them are insoluble in water, and present low solubility in oils and organic solvents [5,6,7]. However, the use of this kind of solvents in encapsulation processes could compromise its subsequent application in food or pharmaceutical products due to its toxicity, low solubilization power strength for these compounds, which generates low loading capacities, or could not be a healthy option, taking into account the obesity epidemic [8]. The encapsulation of β-carotene using organic solvents or oils in different biopolymeric matrices was extensively reviewed by Soukoulis and Bohn [9].

A promising alternative is the use of deep eutectic solvents (DES). DES consist of an eutectic mixture of two (or more) components, namely a hydrogen bond acceptor (HBA), such as quaternary ammonium halide salts and metal chloride, and a hydrogen bond donor (HBD), such as alcohols, amides, and carboxylic acids, which are able to self-associate to lead to a liquid temperature of their mixture significantly lower than the pure equivalents [10,11]. This new kind of solvents (DES and natural deep eutectic solvents (NADES)) has gained considerable attention due to their unique physicochemical properties, such as negligible volatility, liquid state far below 0 °C, broad range of polarity, high solubilization power strength for a wide range of compounds, especially poorly water-soluble compounds, high extraction ability, and high stabilization ability for some natural products [12,13]. These unique properties together with their relatively low cost, possible biodegradability, and lower toxicity make DES an excellent replacement for traditional organic solvents [14]. Moreover, a kind of DES called natural deep eutectic solvents (NADES) exists, which are solely based on natural substances, such as organic acids, amino acids, and sugars; this also makes them interesting in health-related areas such as pharmaceuticals, food, and cosmetics [15].

Although DES were first reported in the 1990s, the number of applications explored is still scarce [16]. DES have been taken into consideration mainly as extraction media, and as solvents in biocatalytic and enzymatic processes, but some research work related to food and pharmaceutical applications has also been performed. One of the first reported examples related to food or pharmaceutical applications was the use of DES as drug vehicles for transdermal delivery of pharmaceutical compounds [17]. After that, some examples appeared using DES as solvents in conventional encapsulation processes, such as the encapsulation of camptothecin, lidocaine, or folic acid by in situ polymerization [18,19,20,21]; the encapsulation of anthocyanins in calcium alginate [22]; and the loading of curcumin into a hydrophilic hydrogel [23]. However, conventional encapsulation processes require the use of high temperatures, toxic reactives, or oxygen permeable polymeric matrices, which could affect the bioactivity of the bioactive compound, or compromise its application in a food or pharmaceutical product.

Electrohydrodynamic spraying or electrospraying is a simple and highly versatile method of liquid atomization by means of electrical forces which allows the production of capsules in the micro, submicro, and nano range [24]. In this process, the liquid flowing out of a capillary nozzle, within a high electrical potential, is forced by the electrostatic forces to be dispersed into fine droplets, which after drying, generate the capsules at room temperature [25,26]. This technology is very suitable for the encapsulation of bioactive compounds due to its mild operating conditions. However, the majority of the bioactive ingredients of interest are not readily soluble in water. In those cases, emulsion electrospraying may be a plausible strategy, in which the bioactive is solubilized into the dispersed phase of an oil-in-water (O/W) emulsion and the encapsulating biopolymer is solubilized in the aqueous phase [27]. In addition, the use of the emulsion allows obtaining particles with narrow size distribution, core-shell structures, and high retention of the bioactive compound [28]. This approach has been employed so far for the encapsulation of a wide number of bioactive compounds within a wide variety of biopolymer matrices [29], including the encapsulation of carotenoids [30,31]. Up to our knowledge, DES have never been applied in the encapsulation of bioactive compounds by electrospraying. The only work related to electrohydrodynamic processing and DES was reported by Mano et al. for the encapsulation of NADES in polymeric fibers by electrospinning [32,33].

The objective of this study is to evaluate the possibility of increasing the stability and loading capacity of β-carotene in WPC capsules, by using DES as solvents and emulsion electrospraying as the encapsulation technique. β-carotene is a very well-known model bioactive compound with low solubility, and many works have been published before in the group dealing with the stabilization of β-carotene [30,34,35,36]. In this study, we investigated the possibility of generating emulsions with these solvents, using an aqueous solution of WPC as a continuous phase, and DES with or without bioactive, as the dispersed phase. The capsules were prepared by emulsion electrospraying and were characterized according to their morphology and loading capacity. The infrared spectrum of the capsules was studied to confirm the presence of DES within the capsules. Finally, a study of stability against photo-oxidation was performed.

## 2. Results and Discussion

### 2.1. Characterization of the Emulsion

First, a physical characterization of the emulsions was performed since it is well-studied that the morphology of electrosprayed materials highly depends on the physical characteristics of the initial polymeric solutions, such as viscosity, surface tension, and electrical conductivity. For instance, the viscosity should be in optimum value in order to form the encapsulation structures from polymer entanglements [37]. The surface tension should be low enough to form a stable Taylor cone that leads a stable electrospraying process [38]. Lastly, the electrical conductivity should not be too high in order to achieve stable electrospraying [39,40].

Initially, different concentrations of WPC were tested in order to obtain a successfully sprayable solution and it was found that a concentration of 30% (*w*/*v*) WPC solution was optimum for the electrospraying process. For a concentration of 20% (*w*/*v*) only drops of material were obtained on the collector. On the other hand, at a concentration of 40% (*w*/*v*) a gelled structured was obtained (results not shown). It was concluded that the optimum concentration for WPC capsule formation was 30% and, thus, this concentration was used for the rest of the experiments. Thereafter, different emulsion systems were characterized before producing the encapsulation structures. Table 1 shows the prepared O/W emulsions, with solutions of WPC (30% *w*/*v*) as continuous phase, and DES (2%, 10% *v*/*v*) as the disperse phase. For all cases, 5% wt. Span 20 surfactant was used.

Results in Table 1 show that the average viscosity of the WPC solution was increased with the incorporation of DES from 61.8 cP to 101.2 cP. However, no significant differences were observed when increasing the percentage of DES from 2% to 10% in the emulsions. The surface tension of all the systems was low enough for electrospraying, as the values did not exceed the limit of 50 mN/m which was suggested previously as the limit for electrospraying [41], thanks to the use of the surfactant. The electrical conductivity values increased with the addition of DES. Most particularly, the conductivity values increased approximately 212% due to the increase in the DES concentration from 2% to 10%. Furthermore, the addition of antioxidant, β-carotene, decreased the conductivity 21.8% on average, due to the well-known low polarity of the bioactive.

### 2.2. Morphology

**Figure 1:**
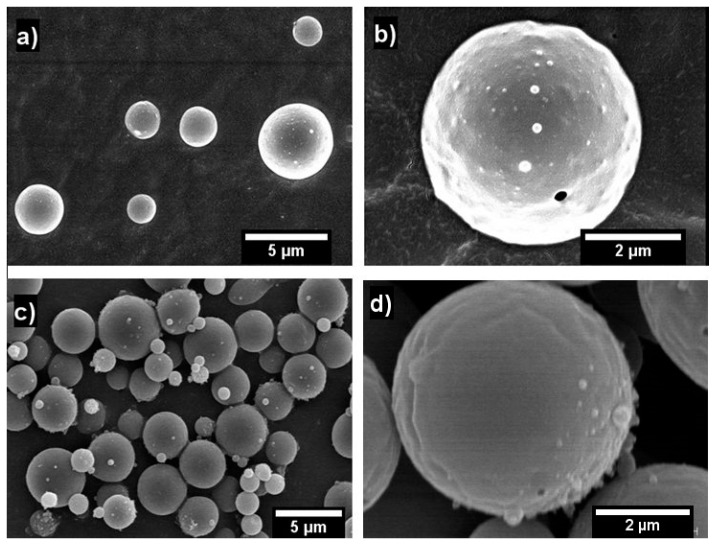
Scanning electron microscopy (SEM) images of the electrosprayed capsules of: (**a**) 30% WPC + 10% DES4 with scale marker of 5 µm; (**b**) 30% WPC + 10% DES4 with scale marker of 2 µm; (**c**) 30% WPC + 10% DES4 + β-carotene with scale marker of 5 µm; (**d**) 30% WPC + 10% DES4 + β-carotene with scale marker of 2 µm.

SEM images shown in Figure 1 demonstrate that round, smooth WPC capsules are obtained through electrospraying from WPC + 10% DES4 system. As seen from the figure, β-carotene-containing WPC capsules (Figure 1c,d) exhibited similar morphology with the non-β-carotene-containing WPC capsules (Figure 1a,b). Furthermore, these particles were similar in size to those obtained in previous work for WPC/glycerol systems [30]. Mean capsule diameters were 2.56 μm (Figure 1c,d) and 2.51 μm (Figure 1a,b) for the neat WPC and β-carotene-containing WPC capsules, respectively. The here-observed capsule diameters were relatively high despite the low surface tension values (Table 1), which are normally considered as a factor to influence the morphology of electrospun systems [30]. Therefore, other factors like solution properties (e.g., conductivity, viscosity) and processing parameters, including polymer degradation, may be playing a major role in determining capsule size [42].

Since DES and surfactant do not show volatility and the surface morphology of the particles exhibit some roughness with spherical features, it is expected that the β-carotene dissolved in DES will be homogeneously distributed in nanovesicles within the microparticles, as seen in previous works by the same authors [43,44]. The fact that the DES and surfactant remain inside the capsule should not bring a problem in commercial formulations, since all the components are currently being used as additives in foods, and in addition, they could promote absorption at the gastrointestinal tract [45].

### 2.3. Loading Capacity

It is well-known that β-carotene is a bioactive compound with a low bioavailability due to its accused lipophilicity; indeed, only 20% of the β-carotene consumed is absorbed in the gastrointestinal tract [9], as the recommended daily dose of β-carotene for an adult is between 1.6 and 9.6 mg [46]. In order to estimate the daily dose intake, it is essential to calculate the loading capacity of the capsules.

The loading capacity of the capsules produced with a concentration of 2% NADES/DES was relatively low (6.67 × 10^−3^%max, 0.0667 × 10^−3^%min). The highest β-carotene loading capacity was calculated for the WPC emulsion with 10% DES4 (37 × 10^−3^%) since the initial concentration of β-carotene in DES4 was the highest (1000 µg/mL). This loading capacity entails that a consumption of 4 g of capsules is required to obtain the recommended intake. Previous studies, such as the work of Rodrigues et al., reported very little loading capacities, meaning that up to 100 g of capsules may be required to get the recommended daily intake [31]. In a previous study carried out in a lab by López-Rubio et al. [30], a loading capacity of 79.3 × 10^−3^% was obtained via the encapsulation in WPC of a suspension of β-carotene particles in glycerol (results shown in Table 2). The obtained loading capacity by López-Rubio et al. [30] would then require a potential consumption of 2 g of capsules. However, since bioavailability is known to be significantly better in solution [47], rather than in particles suspension, the use of DES can thus help improve the bioavailability of β-carotene. Hence, this emulsion was used for further experiments.

### 2.4. Infrared Spectroscopy

**Figure 2:**
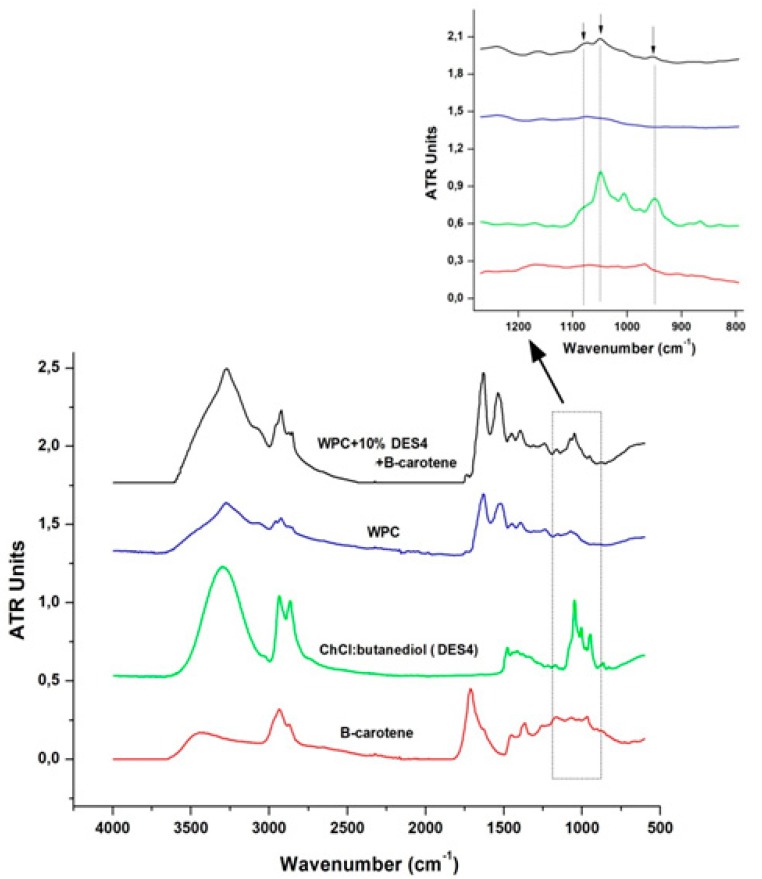
ATR-FTIR spectra of electrosprayed capsules. From bottom to top: pure β-carotene crystals, ChCl:butanediol (DES4), pure WPC, and WPC + 10% DES4 + β-carotene capsules. The arrows point to the spectral band related to the presence of ChCl:butanediol (DES4).

ATR-FTIR spectroscopy was performed to analyze the obtained capsules and verify the presence of the DES containing β-carotene in the capsules produced. Figure 2 shows the FTIR spectra of pure WPC, ChCl:butanediol (DES4), pure β-carotene crystals, and WPC + 10% DES4 + β-carotene capsules. The ATR-FTIR spectrum of the β-carotene-loaded encapsulate shows contributions from both the protein wall material and the DES4 solvent containing β-carotene. The characteristic bands of proteins (i.e., Amide I (ν C=O, ν C–N) at 1650 cm^−1^ and Amide II (δ N–H, ν C–N) at 1550 cm^−1^) were clearly discerned in the spectrum of the β-carotene-loaded WPC encapsulate [48]. The β-carotene-loaded encapsulate also showed a considerable relative increase in the absorbance of the bands between 2800 and 3600 cm^−1^ with respect to the pure protein, assigned to the contribution of the DES4 solvent. Thus, the bands between 3000–3600 cm^−1^ are attributed to DES solvent N–H and O–H groups [49], responsible for forming hydrogen bonds with carbonyl groups of the peptides in the proteins [50]; and the bands between 2800–3000 cm^−1^ are assigned to DES solvent C–H stretching vibrations of carbonyl groups [51]. In addition, the ATR-FTIR spectrum of the encapsulate containing β-carotene shows a new peak at 1080 cm^−1^, which corresponds to the C–O stretching peaks of the primary alcohol in ChCl:butanediol components in DES4 [52]. As seen from Figure 2, no characteristic peaks of β-carotene were detected in the ATR-FTIR spectrum of the encapsulate containing β-carotene. This is most likely due to the low concentration of β-carotene in the capsules, whose contribution would be masked by the intense peaks of the WPC. However, the presence of the DES bands suggests indirectly the presence of β-carotene, since the carotenoid is dissolved in the solvent. In any case, the presence of β-carotene in the capsules was confirmed by UV/Vis spectrophotometry as shown in the following section.

### 2.5. Stability of the WPC Capsules Containing β-Carotene Against Photooxidation

**Figure 3:**
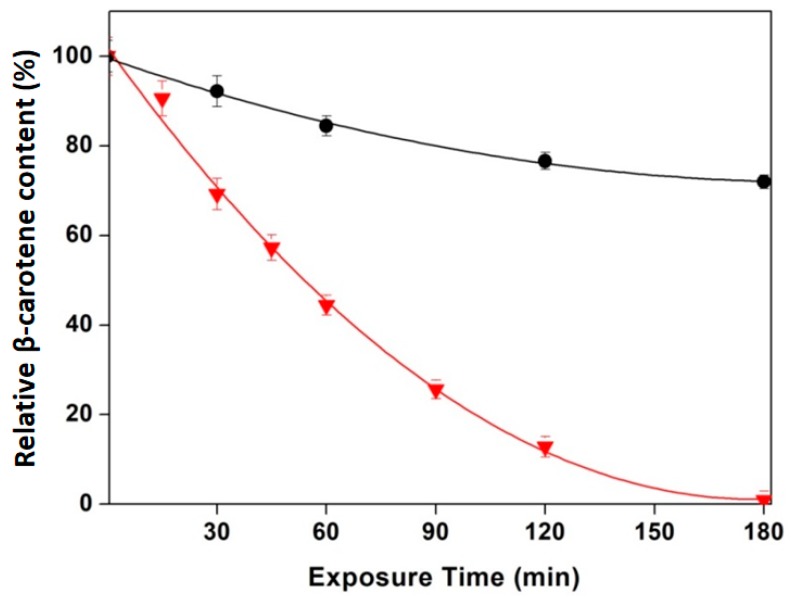
Relative photo-oxidation vs. UV/Vis exposure time in the β-carotene concentration measure by UV/Vis spectroscopy at 448 nm of (●) as WPC + 10% DES4 + β-carotene capsules, (▼) free β-carotene.

The confirmation of the presence, and subsequent quantification, of β-carotene in the capsules was done by resolubilization of these for the stability study after UV exposure. Thus, a comparison of the photostability between β-carotene in hexane and β-carotene microencapsulated in WPC was carried out and can be seen in Figure 3. At the same time, we checked that the efficiency of the encapsulation process was 100%, since all β-carotene in the solution was found trapped in the capsules. β-carotene is a highly light-sensitive molecule due to the double bonds in the molecule [35] and its photoisomerization is often considered as being able to occur in solution, hexane in this case, but not very readily in dry particles [53]. As observed in the figure, the β-carotene loaded WPC capsules showed very good photo-oxidation stability. After 180 min (3 h) of light exposure, complete degradation of the free β-carotene in the hexane solution was measured, whereas 72% of the encapsulated β-carotene remained stable in the same time interval.

## 3. Materials and Methods

### 3.1. Materials

Whey protein concentrate (WPC) was purchased from Davisco Foods (Le Sueur, MN, USA) and was used without further purification. The composition per 100 g of product consisted of ~80 g of protein, ~9 g of lactose, and ~8 g of lipids, the rest being water and minerals. The surfactant (Span20), synthetic β-carotene ≥93%, 1,4 butanediol 99% (ReagentPlus^®^), choline chloride ≥98% (ChCl), glycerol ≥99%, and glucose were purchased from Sigma-Aldrich (St. Louis, MO, USA). Distilled water was used throughout the study. N-hexane (98% purity) was purchased from Aklar Kimya (Ankara, Turkey). Ultra-Vitalux (300 W) lamp was purchased from OSRAM (Munich, Germany).

### 3.2. Preparation of NADES and β-Carotene Solutions

Four different deep eutectic solvents, NADES1 (ChCl:propanediol:water), NADES2 (ChCl:Glucose:water), NADES3 (ChCl:Glycerol), and DES4 (ChCl:butanediol), were prepared by weighting the components in proper mole ratios, respectively 1:1:1, 5:2:5, 1:2, and 1:2. The mixtures were heated under stirring at 50 °C until a clear liquid was obtained. The mixtures remained in the liquid state after cooling to room temperature. Then, solutions of β-carotene in NADES1 (250 µg/mL), NADES2 (10 µg/mL), NADES3 (100 µg/mL), and DES4 (1000 µg/mL) were prepared, corresponding to the highest concentration of β-carotene that can be attained in those mixtures at room temperature. It should be noted that β-carotene is insoluble in ethanol, glycerol, and propylene glycol; slightly soluble in boiling organic solvents such as ether (0.05%), benzene (0.2%), carbon disulfide (1%), and methylene chloride (0.5%); its solubility in edible oils is of 0.08% at room temperature, 0.2% at 60 °C, and 0.8% at 100 °C [54]. The calculated solubility of β-carotene in DES4 is of ca. 0.08%, similar to that of oil at room temperature.

### 3.3. Preparation of WPC Solutions

WPC solutions were prepared by dissolving a precise amount of the material in distilled water through gentle stirring at room temperature in order to achieve three different WPC concentrations of 20%, 30%, and 40% (*w*/*v*). These solutions were used first to prepare the single WPC capsules. For all cases, 5 wt.% of Span 20 surfactant was used to facilitate the electrospraying process. In case of emulsions, β-carotene solubilized in the four different DES solutions was added to the aqueous WPC solutions to attain a final 2% and 10% (*v*/*v*) concentrations. The resultant mixture was homogenized using a TX4 Digital Vortex Mixer from Velp (Usmate, Italy) to generate an emulsion.

### 3.4. Electrospraying Process

The electrospraying apparatus used was a Fluidnatek LE-50 Capsultek^®^ tool from Bioinicia S.L. (Valencia, Spain) in the lab mode configuration, with a single needle injector placed horizontally towards a grounded flat collector. The equipment has a variable high-voltage 0–30 kV power supply, and a 5 mL plastic syringe was used as a reservoir for the solution. The distance between the needle and the collector was set at 15 cm. The capsules were obtained using a voltage range of 17–23 kV and a flow rate range of 50–100 µL/h. The operation parameters were optimized for each formulation to get a stable cone of Taylor, which ensures stable processing. The particles were collected dry and no further drying procedure was applied. The efficiency of the process, in terms of the bioactive present in the collector vs. that in the starting solution, was found to be of 100% for the lab scale system used.

### 3.5. Characterization

#### 3.5.1. Characterization of the Solutions and Emulsions

Emulsions and solutions were characterized in terms of surface tension, viscosity, and conductivity. The surface tension was measured using the Wilhemy plate method in an EasyDyne K20 tensiometer (Krüss GmbH, Hamburg, Germany). The viscosity was measured using Visco Basic Plus (Fungilab, Barcelona, Spain). The conductivity measurements were carried out using a HI98192 conductivity probe (Hanna Instruments, Gothenburg, Sweden). All the measurements were performed at room temperature.

#### 3.5.2. Morphological Characterization

The morphology of the capsules was examined by scanning electron microscopy (SEM). The SEM micrographs were taken using a Hitachi S-4800 electron microscope (Tokyo, Japan) at an accelerating voltage of 10 kV and working distance of 8 mm. A few milligrams of the collected powder were deposited over a double side carbon strip prior to sputtering with a gold-palladium mixture for 3 min under vacuum. The average capsule diameter was determined via ImageJ Launcher software program from the SEM micrographs in their original magnification.

#### 3.5.3. Loading Capacity of Whey Protein Concentrate (WPC) Capsules

Weight percent loading capacity (LC) was calculated on the basis of the mass of β-carotene over the mass of WPC capsules containing β-carotene, in duplicate. It can be represented as:

$$LC\;\left(\%\right)=\frac{Mass\;of\;\beta -carotene}{Mass\;of\;\beta -carotene+Mass\;of\;WPC+Mass\;of\;Surfactant+Mass\;of\;DES}\cdot 100\%$$

#### 3.5.4. Infrared Spectroscopy

Attenuated total reflectance infrared spectroscopy (ATR-FTIR) experiments were performed using a Bruker FTIR Tensor 37 equipment (Rheinstetten, Germany). Approximately 50 mg of capsules were placed on top of the diamond crystal and good contact was assured by using the ATR Sampling Accessory low temperature Golden Gate (Specac Ltd., Orpington, UK). All the spectra were obtained within the wavenumber range of 4000–600 cm^−1^ by averaging 10 scans at 4 cm^−1^ resolution. Measurements were performed in triplicate. Analysis of spectral data was carried out using the OPUS 4.0 data collection software program (Bruker, Ettlingen, Germany).

#### 3.5.5. Stability of the WPC Capsules Containing β-Carotene Against Photooxidation

Stability against photo-oxidation of the β-carotene-containing WPC capsules was studied by placing the various samples under UV light at room temperature. For this purpose, the antioxidant degradation rate was compared with that of β-carotene encapsulated in WPC capsules and β-carotene crystals dissolved in hexane which were placed under an Osram Ultra-Vitalux (300 W) lamp in order to accelerate the oxidation of β-carotene over 3 h. This lamp produces an intense mix of radiation very similar to that of natural sunlight. This blend of radiation is generated by a quartz discharge tube and a tungsten filament. The bulb is made of special glass which allows only that part of the output that is contained in natural sunlight to pass through. The distance between the lamp and the samples was 20 cm. Samples were collected over periods of illumination times and the intact β-carotene concentration was determined using T80+ UV/Vis Spectrometer (PG Instruments Ltd., Lutterworth, UK) at 448 nm. The β-carotene concentration of the control samples containing β-carotene crystals dissolved in n-hexane was determined directly by spectrophotometry. For the solutions of β-carotene-loaded WPC capsules, distilled water was added to disrupt the WPC capsules before hexane was added to extract the β-carotene. This two-phase system was generated by the ratio of 1:5 water:hexane composition. This mixture was ultrasonicated for 60 min 20 °C with JY92-IIN Ultrasonic Homogenizer (Hinotek, Ningbo, China) and then centrifuged at 9000 rpm for 3 min. The organic (upper) phase was separated, and the β-carotene concentration was determined by spectrophotometry.

## 4. Conclusions

In this paper, the encapsulation of a bioactive compound by electrospraying using DES as a solvent was screened for the first time. DES are presented as a green alternative for the encapsulation of challenging bioactive compounds due to their low solvation capacity in conventional solvents via the electrospraying encapsulation process. Four different DES formulations were tested, based on mixtures of choline chloride with propanediol, water, glucose, glycerol, and butanediol. First, the formulation of stable O/W emulsions was studied, using WPC aqueous solutions as continuous phase and β-carotene in DES solutions as dispersed phase. Ratios dispersed phase:continuous phase of 2:98 provided stable emulsions with DES formulations containing choline chloride, water, propanediol, glucose, or glycerol. However, DES containing choline chloride and butanediol generated stable emulsions at ratios 10:90, and in this solvent the highest amount of β-carotene was solubilized. With this DES formulation, spherical and smooth β-carotene in a solubilized form loaded WPC capsules with sizes around 2 µm were obtained. Loading capacity in these capsules was the highest for the formulations studied, achieving values in the same order of magnitude of the loading capacity obtained previously using suspensions of β-carotene particles in glycerol. The presence of DES inside the capsules was demonstrated by ATR-FTIR spectroscopy. The accelerated oxidative stability test performed confirmed the protective role of the biopolymer wall. While the free β-carotene oxidized completely within 180 min under UV light, the β-carotene loaded in the WPC capsules only oxidized around 20%. Nevertheless, it would be interesting to explore other NADES formulations and encapsulants that may offer a greater β-carotene solubilization capacity and thus further enhance the loading capacity. The promising results obtained in this work with the combination of DES and the electrospraying processing technique, make the present study of fundamental value in the emerging area of bioactives encapsulation.

## Figures and Tables

**Table 1:** Physical parameters of the studied solutions and emulsions. All samples contain whey protein concentrate (WPC) aqueous solution at a concentration of 30% *w*/*v* and 5% wt. Span 20. DES, deep eutectic solvents; NADES, natural deep eutectic solvents.

Formulations	Viscosity (cP)	Surface Tension (mN/m)	Conductivity (mS/cm)
	**Solutions**		
WPC	61.8 ± 0.8	31.1 ± 0.7	6.0 ± 0.1
	**Emulsions**		
WPC + 2% NADES1	101.2 ± 1.5	31.0 ± 0.5	6.8 ± 0.1
WPC + 2% NADES2	98.6 ± 1.4	32.0 ± 0.5	6.6 ± 0.1
WPC + 2% NADES3	98.3 ± 1.2	32.8 ± 0.7	6.4 ± 0.0
WPC + 2% DES4	100.9 ± 0.9	35.1 ± 1.1	5.9 ± 0.0
WPC + 10% NADES1	91.2 ± 1.2	28.9 ± 0.3	21.1 ± 1.2
WPC + 10% NADES2	97.8 ± 1.7	33.4 ± 0.6	20.0 ± 0.9
WPC + 10% NADES3	99.4 ± 1.3	30.7 ± 0.2	20.8 ± 1.2
WPC + 10% DES4	95.1 ± 0.6	33.0 ± 1.0	19.8 ± 1.3
**Emulsions with Bioactive**			
WPC + 10% NADES3 + β-carotene	95.4 ± 0.7	31.3 ± 0.9	16.0 ± 0.7
WPC + 10% DES4 + β-carotene	93.0 ± 1.3	32.6 ± 0.4	15.7 ± 0.3

**Table 2:** Loading capacity of β-carotene-loaded WPC capsules.

Formulation	Loading Capacity 10^3^ (%)
WPC + 2% NADES1	1.6 ± 0.2
WPC + 2% NADES2	0.1 ± 0.0
WPC + 2% NADES3	0.6 ± 0.0
WPC + 2% DES4	6.2 ± 0.3
WPC + 10% NADES3	3.1 ± 0.1
WPC + 10% DES4	37.0 ± 2.4
López-Rubio et al. [30]	79.3 *

* It was estimated theoretically from the formulation.
